# Differentiation of chromoplasts and other plastids in plants

**DOI:** 10.1007/s00299-019-02420-2

**Published:** 2019-05-11

**Authors:** Najiah M. Sadali, Robert G. Sowden, Qihua Ling, R. Paul Jarvis

**Affiliations:** 1 Department of Plant Sciences, University of Oxford, Oxford, OX1 3RB UK; 2 Present Address: Centre for Research in Biotechnology for Agriculture (CEBAR), University of Malaya, 50603 Kuala Lumpur, Malaysia

**Keywords:** Chloroplast, Chromoplast, Organelle, Plastid, Plastid biogenesis, Plastid protein import, SP1

## Abstract

Plant cells are characterized by a unique group of interconvertible organelles called plastids, which are descended from prokaryotic endosymbionts. The most studied plastid type is the chloroplast, which carries out the ancestral plastid function of photosynthesis. During the course of evolution, plastid activities were increasingly integrated with cellular metabolism and functions, and plant developmental processes, and this led to the creation of new types of non-photosynthetic plastids. These include the chromoplast, a carotenoid-rich organelle typically found in flowers and fruits. Here, we provide an introduction to non-photosynthetic plastids, and then review the structures and functions of chromoplasts in detail. The role of chromoplast differentiation in fruit ripening in particular is explored, and the factors that govern plastid development are examined, including hormonal regulation, gene expression, and plastid protein import. In the latter process, nucleus-encoded preproteins must pass through two successive protein translocons in the outer and inner envelope membranes of the plastid; these are known as TOC and TIC (translocon at the outer/inner chloroplast envelope), respectively. The discovery of *SP1* (*suppressor of ppi1 locus1*), which encodes a RING-type ubiquitin E3 ligase localized in the plastid outer envelope membrane, revealed that plastid protein import is regulated through the selective targeting of TOC complexes for degradation by the ubiquitin–proteasome system. This suggests the possibility of engineering plastid protein import in novel crop improvement strategies.

## Introduction

Approximately 1.5 billion years ago, a mitochondriate eukaryote became host to a cyanobacterium-like prokaryote which eventually became the chloroplast, an organelle wholly dependent on its host cell (Yoon et al. 2004; Archibald 2011). As the monophyletic descendants of a cyanobacterial ancestor, the chloroplasts found in plants and algae today share remarkable similarities with extant cyanobacteria. Chloroplasts belong to the broader plastid family, an important group of plant organelles that are a defining characteristic of the plant cell. In the metaphyta, plastids are able to convert between a number of distinct types during the life cycle of the plant.

During the course of evolution from the endosymbiotic progenitor into the modern chloroplast organelle, the plastid genome (or plastome) became greatly reduced. Many genes were lost completely, as they were no longer needed in the cellular environment, whereas others were transferred to the nucleus (Martin et al. 1998, 2002). The process of gene loss never reached completion, however, as fully functional higher plant chloroplasts today retain about 100 protein-coding genes in their organellar genomes (Martin and Herrmann 1998; Race et al. 1999; Sato et al. 1999). The retained genes are mainly those encoding core proteins of the photosynthesis apparatus, as well as genetic system genes needed to express them, and it has been argued that their presence inside plastids helps to maintain redox balance and overcome the deleterious side effects of photosynthetic electron transport (Race et al. 1999; de Paula et al. 2012; Allen 2015). Most chloroplast proteins are now encoded by nuclear genes, and they must be imported as precursor proteins from the cytosol to reach the inside of the chloroplast.

In addition to the targeting of nucleus-encoded proteins to the plastid, the plastid engages in retrograde signalling to the nucleus. Retrograde signalling is important for coordinating the expression of the nuclear and plastome genes. It involves multiple signalling pathways that relay information from plastids to the nucleus, and it controls most aspects of organelle function including gene expression and development (Nott et al. 2006).

In green algae and lower plants, the plastid continues to serve in its ancestral role of photosynthesis. In higher plants, plastids have taken on roles that extend far beyond photosynthesis, for instance in fruit ripening, endosperm development, and root gravitropism (Waters and Pyke 2005). As well as taking on different forms, they can perform several interrelated roles simultaneously, and the various types of plastid are dynamically interconvertible. The regulated import of nucleus-encoded proteins from the cytosol can lead to major proteome changes in the plastids, and an important mechanism that allows this to occur efficiently involves the remodelling of the translocon at the outer chloroplast envelope (TOC) complexes, which mediate early steps in the protein import process (Jarvis and López-Juez 2013).

The aim of this review is to discuss the factors that may contribute to the differentiation of chromoplasts and other plastids in higher plants, including plastid protein import mechanisms and the recently discovered import regulator, SP1 (suppressor of *ppi1* locus1).

## Plastids are highly dynamic organelles

Plastids are essential for a wide range of normal plant cell functions, and so have a number of specialist forms. Plastids can be divided into several categories based on colour, morphology, and ultrastructure (Whatley 1978; Møller 2005; Wise 2007). The chloroplast is the photosynthetic plastid, and it is named for its green colour. Based on pigmentation, the non-photosynthetic plastids can be broadly divided into leucoplasts, the ‘white’ or colourless plastids, and chromoplasts, the coloured plastids notable for their accumulation of carotenoids. Leucoplasts include the amyloplasts, elaioplasts, etioplasts, and proplastids. The ways in which the chromoplasts may be subdivided and classified are discussed in the next section. A summary of the different plastid types found in plants is presented in Table 1.

**Table 1:** Summary of the different plastid types that exist in plants

Plastid type	Description	References
Amyloplast	Amyloplasts are sites for the synthesis and bulk storage of starch, and are found in roots and storage organs such as cotyledons, seed endosperm, and tubers. They may be involved in gravitropism in the root and shoot apices	Hurkman et al. (2008), Bechtel and Wilson (2003)
Chloroplast	Chloroplasts are found in all photosynthetic tissues and organs. These lens-shaped organelles contain green chlorophyll pigments associated with an internal thylakoid membrane system that mediates the light reactions of photosynthesis	Smillie and Scott (1969), Jarvis and López-Juez (2013)
Chromoplast	Chromoplasts are specialized for the synthesis and storage of high levels of carotenoid pigments, and are commonly found in flowers, fruits, leaves and roots	Camara et al. (1995), Egea et al. (2010), Li and Yuan (2013)
Elaioplast	Elaioplasts are plastids that are specialized for the synthesis of lipids, for example in exine formation during pollen development	Quilichini et al. (2014), Zhu et al. (2018)
Etioplast	Etioplasts are chloroplast progenitor organelles that develop in plants grown in continuous darkness. They rapidly differentiate into chloroplasts upon illumination	Sperling et al. (1998), Plöscher et al. (2011)
Gerontoplast	Gerontoplasts are derived from chloroplasts in senescent leaves. They are characterized by the breakdown of chlorophyll and of the thylakoid membrane system	Matile et al. (1996), Hørtensteiner and Krautler (2011)
Leucoplast	Leucoplasts are colourless plastids found in non-photosynthetic tissues such as endosperm, tubers, roots, and lipid storage organs. Plastids of this type include amyloplasts and elaioplasts	Carde (1984), Howitt and Pogson (2006)
Proplastid	Proplastids are undifferentiated plastids found primarily in meristematic cells and sometimes during egg cell and pollen formation in higher plants. These colourless plastids have no distinctive morphology	Reiter et al. (1994), Waters and Pyke (2005), Pyke (2013)

During plant development, plastids must differentiate appropriately into the required form or type. Proplastids are the plastids found in meristematic tissues, and they possess only primitive internal membrane elements (Liang et al. 2018). During leaf development in the absence of light, proplastids may differentiate into etioplasts with rudimentary internal membranes (including prolamellar bodies and prothylakoids) (Kowalewska et al. 2016); alternatively, in the presence of light, proplastids differentiate into chloroplasts, which accumulate the green pigment chlorophyll and possess a complex internal membrane system called the thylakoids. Moreover, even differentiated plastid types may be interconvertible: for example, etioplasts, when exposed to light, can rapidly redifferentiate into chloroplasts.

In different circumstances, proplastids may give rise to chromoplasts (Ben-Shaul and Klein 1965). Alternatively, chromoplasts may arise from the redifferentiation of chloroplasts in a process which involves chlorophyll degradation occurring in parallel with other ripening events such as cell wall softening and a range of physiological and biochemical changes (Bathgate et al. 1985; Egea et al. 2010).

Different plastid types have distinct proteomes (Balmer et al. 2006; Siddique et al. 2006; Barsan et al. 2010), and so, during plastid transitions, the organellar proteome must become reorganized. This may involve a number of processes, including: extensive reprogramming of gene expression (Deng and Gruissem 1987; Kahlau et al. 2006; Li et al. 2010); differentially regulated import of nucleus-encoded proteins (Bauer et al. 2000; Soll and Schleiff 2004; Jarvis and López-Juez 2013); and the regulated turnover of plastid proteins (Izumi and Nakamura 2018).

Among the non-photosynthetic plastids, amyloplasts and chromoplasts are the most studied because of their relevance to the nutritional qualities of major commodity crops. Amyloplasts are starch-accumulating leucoplasts that are important in agriculture for the nutritional value which they provide in roots (e.g., in cassava) and tubers (e.g., in potato) and in seed endosperm (e.g., the grain of cereal crops). They have principally been studied for their roles in gravitropism, as recently reviewed by Su et al. (2017), and in starch biosynthesis (Dupont 2008; Matsushima et al. 2014).

Chromoplasts function in the synthesis and storage of carotenoid pigments in flowers and fruits, and in certain leaves and roots. The colouration of petals by chromoplasts is an evolutionary strategy adopted by some angiosperms to attract pollinators (Waters and Pyke 2005; Egea et al. 2010, 2011). As animal pollination is increasingly being identified as a limiting factor in intensive farming, this may become of agricultural relevance (Winfree 2008). Furthermore, the differentiation of chromoplasts in fruit ripening, for example in tomato or bell pepper, is an important part of the ripening process, and so is relevant to the timely harvesting and subsequent distribution of fruit (Li and Yuan 2013; Pesaresi et al. 2014). As carotenoids have nutritional value, they also affect food quality and there is interest in enhancing carotenoid biosynthesis in crops (Taylor and Ramsay 2005).

## Chromoplast structure and biochemistry

### Chromoplast morphology

Among the early processes that occur during the chloroplast-to-chromoplast transition, the most conspicuous are changes in biochemical composition (e.g., the elevation of carotenoid and quinone contents) and structural organization (e.g., fibril assembly and degradation of thylakoids) (Ljubesic et al. 1991). Early work on chromoplast biology and the categorization of different types of chromoplasts in different tissues and species were largely conducted by light microscopy (Harris and Spurr 1969; Møller 2005). Our present understanding of chromoplast development and transitions owes much to the development of techniques for the isolation of intact chromoplasts and the advancement of microscopic methods (Camara et al. 1995).

With the advent of electron microscopy, greater resolution of chromoplast morphologies related to pigment storage became possible, and distinct internal substructures could be categorized (Devidé and Ljubešić 1974; Camara et al. 1995; Waters and Pyke 2005). Chromoplast classification is based on the frequency of such substructures within the organelle. For example, globular chromoplasts are characterized by the accumulation of plastoglobules containing pigments in the stroma, and these are typically found in pumpkin fruit. The plastoglobules in chromoplasts are distinguishable from those in chloroplasts and gerontoplasts; for example, plastoglobules observed in mature chloroplasts are much smaller than those in chromoplasts, whereas those in gerontoplasts contain more xanthophyll esters, which are formed during breakdown of carotenoids (Lichtenthaler and Weinert 1970; Mulisch and Krupinska 2013).

In fact, Camara et al. (1995) classified several types of chromoplast in different tissues and plant species. Crystalline chromoplasts accumulate crystals of lycopene or β-carotene and are typically found in the chromoplasts of tomato fruit. Membranous chromoplasts, typically found in daffodil and ornamental hybrids called florists’ slipperwort, contain extended concentric membranes and a low plastoglobule content. Reticular–tubular chromoplasts contain a complex network of twisted fibrils filling the stroma, together with a few plastoglobules, and are commonly found in tulip. Chromoplasts in the fibrillar and tubular classes contain an extensive microfibrillar network that is organized into bundles or dispersed substructures.

It should be noted that more than one type of substructure may be found within any given chromoplast (Ljubesic et al. 1991; Camara et al. 1995). It may be the case that these structures develop successively, or simultaneously. Pigment content varies in correlation with the structure types present in the chromoplasts, but it is not yet clear what the causal relationship is in this.

### Varieties of chromoplast

Chromoplasts in different tissues or species may represent different sub-types of plastid with different specialisms. A comparative study of chromoplast proteomes from six different crops identified that while there were some broad commonalities (e.g., up-regulation of enzymes involved in carotenoid biosynthesis), the organelles of the different species each had distinct protein abundance profiles (Wang et al. 2013). However, there is a lack of information concerning proteome differences between chromoplasts in different organs of one species (e.g., between tomato fruit and flower), or between chromoplasts that have arisen from different transitions (e.g., chromoplasts that arose directly from proplastids versus those derived from chloroplasts). The proteome of tomato fruit chromoplasts retains significant parts of the photosynthetic apparatus, including Calvin cycle enzymes and photosystem components (Barsan et al. 2010; Suzuki et al. 2015), but it remains to be seen how much of this is functional or merely leftover from the chloroplast progenitors, and whether chromoplasts that have arisen from amyloplasts or proplastids also show such similarities with chloroplasts. Until a more thorough understanding of the molecular differences between chromoplasts from different plant organs and species has been obtained, it is important to bear in mind which system a given study is investigating to avoid generalizing unduly. Therefore, in the rest of this section, observations about chromoplasts have been organized by the system in which they were observed—chromoplasts in non-green leaves, in flowers, and in fruit.

The overlap between the chloroplast and chromoplast proteomes, and the absence of chromoplasts in many evolutionary lines, has been taken as evidence that chromoplasts are the most recently evolved plastid type (Kuntz and Rolland 2012). As *Arabidopsis* does not normally develop chromoplasts, several different experimental systems have been employed to investigate this plastid type.

#### Non-green leaves

Non-green colouration of plant leaves may or may not represent chromoplast differentiation. For example, during senescence, leaves lose their green colouration and become yellow or brown. This represents chlorosis and the transition from chloroplasts to gerontoplasts, where green chlorophylls are degraded and partially retained carotenoids are revealed (Falk 1976; Matile et al. 1996), and is not the transition from chloroplasts to chromoplasts. The carotenoids found in senescent leaves are typically not newly synthesized in gerontoplasts: in this respect, gerontoplasts are different from chromoplasts in fruits and flower petals, despite the fact that both organelle types develop from chloroplasts (Matile et al. 1996).

An interesting case is that of the common box tree, the leaves of which become red during autumn and winter due to the de novo synthesis of red carotenoids (Hormaetxe et al. 2004). This is a response to photoinhibitory conditions during winter acclimation, and is reversed on exposure to warmer temperatures (Hormaetxe et al. 2004). Structurally, the chloroplasts are reorganized to form the typical globular chromoplasts, and can be restored in shape, colour, and size into chloroplasts again in warmer weather (Koiwa et al. 1986). This provides an interesting example of reversible chromoplast differentiation based on environmental cues.

Red colouration in leaves may also come from the accumulation of anthocyanin in the vacuoles of epidermal tissue. This is thought to be a protective measure against high light conditions or insect herbivory (Karageorgou and Manetas 2006), and it is linked to impaired rates of carbon assimilation (Gould et al. 2002). As the plastids still carry out photosynthesis, albeit at reduced rates, they may still be characterized as chloroplasts.

#### Flower chromoplasts

Chromoplasts in petals generate pigments that colour flowers and encourage pollinators. In many species, for example wallflower, young petals dissected from unopened buds contain chloroplasts containing chlorophyll throughout their structure, and these must redifferentiate into chromoplasts (Weston and Pyke 1999). The system of differentiation from chloroplasts to chromoplasts in petals is more similar to that in fruit chromoplast differentiation than to that in leaf chromoplast differentiation, as chloroplasts from leaves contain more stromal thylakoids and grana than chloroplasts found in immature green fruits and petals (Marano et al. 1993).

In *Arabidopsis*, where the petals are white, the chloroplasts of the immature petals differentiate into colourless leucoplasts. During this transition, the plastids degrade chlorophyll, but do not produce carotenoids, presumably caused by a failure to initiate carotenoid synthesis. This results in the formation of a mature petal with a white blade and a stalk that remains green owing to the persistence of chloroplasts (Pyke and Page 1998). In white petals of chrysanthemum, carotenoid cleavage dioxygenase (CCD4a) protein was identified to be the factor that inhibits the accumulation of carotenoids, which results in the white colour (Ohmiya et al. 2006).

In some species, chloroplasts that are capable of performing photosynthesis are maintained in specific cell types within mature petals (Vainstein and Sharon 1993; Weston and Pyke 1999). For example, the chlorophyll-containing chloroplasts in the mature petunia corollas appear to be confined to the interior mesophyll tissue, whereas non-green pigment is confined to the epidermal cells (Waters and Pyke 2005). During chromoplast differentiation in the petals of watercress (Falk 1976) and cucumber (Smith and Butler 1971), the first noticeable changes are the degradation of the thylakoid membranes in the chromoplasts of the unfolding petals, and the process ends with the complete absence of internal membranes in the plastids once the stage of an open flower is reached.

With modern molecular and cytological techniques, there is much potential for greater exploration, understanding, and manipulation of chloroplast-to-chromoplast differentiation in petals. For example, such work may elucidate the molecular events that occur during carotenoid biosynthesis in petals (Ohmiya 2013), or achieve alteration of flower colour (Yuan et al. 2015). Comparing patterns of carotenoid accumulation and the expression of the corresponding biosynthetic genes during petal development of morning glory proved informative (Yamamizo et al. 2009), while the modification of model legume, *Lotus japonicus*, by the overexpression of a *crtW* gene, encoding β-carotene ketolase, altered flower colour from yellow to orange owing to altered accumulation of ketocarotenoids (Suzuki et al. 2007).

#### Fruit chromoplasts

The global demand for fruit has resulted in extensive research to elucidate the biochemical and molecular mechanisms of carotenoid biosynthesis and chromoplast differentiation during fruit ripening (Prasanna et al. 2007). Fruit chromoplasts are frequently studied in yellow, orange, and red fruits including tomato, orange (citrus), and bell pepper.

The transition of chloroplasts into chromoplasts represents one of the most visible events in fruit ripening. The most apparent changes are the degradation of chlorophyll, the disruption of the thylakoids, and extensive synthesis of carotenoid pigments (Grierson and Kader 1986; Marano et al. 1993; Deruère et al. 1994; Prasanna et al. 2007). Analysis of the *green flesh* (*gf*) mutant of tomato revealed that the breakdown of chlorophyll-containing thylakoid membranes and the formation of new chromoplast membranes are separate, independent processes (Cheung et al. 1993). In the *gf* mutant, detectable amounts of chlorophyll and many thylakoid stacks remained as the fruits ripened. However, the *gf* mutation had no negative effect on the formation of chromoplast membranes or the accumulation of carotenoids. The defect in chlorophyll degradation and unimpaired chromoplast development in the mutant fruits suggest that chromoplast differentiation is an independent and defined developmental process rather than merely the uncontrolled breakdown of chloroplast structure (Cheung et al. 1993). The *gf* mutation and *chlorophyll retainer* (*cl*), a pepper mutation that also perturbs the turnover of chlorophyll, both affect homologues of the rice *STAY*-*GREEN* (*SGR*) gene, originally identified for its role in chlorophyll turnover during senescence (Barry et al. 2008).

The SGR protein is a chloroplast-targeted, senescence-associated protein (Barry et al. 2008; Gapper et al. 2013). Although *SGR* family genes are known to function in chlorophyll degradation, and *SGR*-like genes are found consistently across the higher plants, their roles seem to vary somewhat in different species (Sakuraba et al. 2015). In tomato fruit ripening at least, SGR (GF) is additionally involved in the induction of ethylene signalling (ethylene’s role in ripening is discussed in more detail later), and, surprisingly, it interacts directly with the carotenoid synthesis enzyme phytoene synthase 1 (PSY1) (Luo et al. 2013).

Most studies have characterized fruit chromoplast differentiation in two different systems—those that can or cannot be reversed. The ripening of citrus fruit (Iglesias et al. 2001; Alós et al. 2006), pumpkins (Devidé and Ljubešić 1974; Schaffer et al. 1984), and cucumber fruit (Prebeg et al. 2008) is considered to involve the reversible differentiation of chromoplasts. As in the common box tree example described earlier, the mature chromoplasts in these fruits can redifferentiate into chloroplasts with normal structure and photosynthetic capacity.

Regreening involves the elevation of chlorophyll content and the reduction of carotenoid content, and this can be promoted by excess nitrate and gibberellin (e.g., by application to the fruit on the tree). A high abundance of nitrogen in the pericarp tissue retards the chloroplast-to-chromoplast transition and promotes regreening in citrus fruit (Huff 1984). The effect of nitrogen appears to have a protective role in stabilizing chloroplast structure, and diminishes the effect of high sugar levels in inducing transformation of chloroplasts to chromoplasts, rather than promoting chloroplast formation from chromoplasts (Huff 1984). Such on-tree treatments of citrus fruit were found to alter gene expression: Gibberellin and nitrate treatments inhibited the expression of genes for regulatory enzymes of carotenoid biosynthesis [e.g., 1-deoxy-d-xylulose 5-phosphate synthase (DXS)] and chlorophyll degradation [e.g., pheophorbide *a* oxygenase (PaO)] (Alós et al. 2006), and, consequently, delayed colour breaks (defined as an increase in carotenoid content and a reduction in chlorophyll content) in the fruit (Alós et al. 2006). These observations suggested a mechanistic basis for the regulation of colour breaks and regreening in citrus fruits. Such regreening has an economic impact, because the fruits, although internally mature, are not marketable due to the reduction of sugar levels (Iglesias et al. 2001).

In contrast, regreening has not been observed in other species like tomato and pepper, where chromoplast development is regarded as terminal, possibly due to irreversible structural breakdown of the thylakoids. In tomato and pepper fruit, chromoplasts are derived from fully developed chloroplasts in the fruit tissue (Bathgate et al. 1985; Cheung et al. 1993; Egea et al. 2011; Gapper et al. 2013; Li and Yuan 2013). Egea et al. (2010) visualized the transition from chloroplast to chromoplast in a purified plastid fractionation experiment in tomato. The transition can be visualized by exploiting the different autofluorescence emissions of the chlorophyll (taking measurements at 740–750 nm) and carotenoid (500–510 nm) pigments that characterize the different plastid types. In another microscopy study of tomato fruit ripening, the intermediate yellow colour of semi-ripe tomatoes was found to be due to the presence of both chloroplasts and chromoplasts in the same tomato tissue (Camara et al. 1995).

The proteomics of chromoplast differentiation in tomato was explored in a comparative study of plastids at three stages of fruit ripening (mature-green, breaker and red) (Barsan et al. 2012). This revealed metabolic shifts that are coupled with the down-regulation of the thylakoid biogenesis machinery and the up-regulation of carotenoid biosynthesis components; the changes included decreases in carbohydrate metabolism (starch synthesis and degradation) and in reactions that occur in the light (photosynthesis, including the Calvin cycle, and photorespiration), and increases in stress-response proteins (redox, heat shock, and ascorbate–glutathione cycle) and terpenoid biosynthesis enzymes (required for carotenoid synthesis) (Barsan et al. 2012).

Chromoplasts also arise from other non-photosynthetic plastid types, such as proplastids, leucoplasts, or amyloplasts, during fruit ripening or root development. This is known to occur during the development from colourless tissues of carotenoid-enriched tissues in melon, watermelon, papaya, mango, carrot, and sweet potato (Giuliano and Diretto 2007; Li and Yuan 2013).

### Chromoplast biochemistry

#### Carotenoids

The major function of the chromoplast as a specialized storage site is to accumulate the high levels of colourful pigments in plant tissues or organs. The pigments that accumulate in chromoplasts are mostly members of the carotenoid family, including β-carotenes, lycopene, lutein, violaxanthin, and neoxanthin (Camara et al. 1995; Cunningham and Gantt 1998; Lu and Li 2008). Some carotenoids can be converted into others through a variety of complex reactions. During fruit ripening when chloroplasts transform into chromoplasts, the carotenoids are actively synthesized concomitantly with a decrease in chlorophyll levels (Klee and Giovannoni 2011). Tomato provides the principal dietary source of lycopene and a major source of β-carotene, both of which have been highlighted as important food compounds beneficial to human health. Thus, it is of interest to enhance the carotenoid content and profile of tomato fruit for agricultural applications.

#### Lipids

One of the prominent changes during chromoplast differentiation is the remodelling of the internal membrane system to develop carotenoid-lipoprotein sequestration substructures and plastoglobules in chromoplasts. Such structures contain specific lipoprotein fibrils to store the accumulated carotenoid pigments, and it is these that largely define the different types of chromoplasts discussed earlier (Deruère et al. 1994; Egea et al. 2010).

A large number of proteins and key enzymes involved in lipid metabolism and fatty acid biosynthesis were identified in chromoplast proteomes from various crop species, which is indicative of the ability of chromoplasts to synthesize fatty acids and various types of lipids that contribute to the reorganization of the internal membrane networks in the organelles (Wang et al. 2013). For example, the key enzymes involved in lipid catabolism and lipid homeostasis, including AMP-dependent synthetase and ligase, long-chain fatty acid acyl-CoA synthetase, phospholipase Dα1, and multifunctional protein 2 were repeatedly detected in chromoplasts from six carotenoid-rich crops: watermelon, tomato, carrot, orange cauliflower, red papaya, and red bell pepper (Wang et al. 2013).

#### Carbohydrates

A positive correlation of sugar levels with carotenoid accumulation, isoprenoid metabolism, and chromoplast formation has been observed in some studies (Iglesias et al. 2001; Télef et al. 2006; Horner et al. 2007; Flores-Pérez et al. 2010). For example, during amyloplast-to-chromoplast conversion in the developing tobacco nectary, chromoplast differentiation is associated with the production of nectar sugars as well as starch catabolism (Horner et al. 2007). In fact, other studies suggested that carbohydrates, such as sucrose and hexose, could regulate chromoplast differentiation. Evidence showed that sucrose stimulates and promotes the conversion of chloroplasts into chromoplasts, whereas sucrose limitation reverses the process (Iglesias et al. 2001). The role of sugars in controlling carotenoid accumulation may be related to the differential regulation of carotenoid biosynthesis genes. For example, sucrose depletion specifically influences the expression of *PSY1,* a gene controlling synthesis of phytoene (an intermediate of carotenoid biosynthesis), and so can delay carotenoid accumulation in tomato fruit pericarp (Télef et al. 2006). However, other aspects of chromoplast differentiation such as chlorophyll degradation and starch catabolism, are apparently not affected by sucrose availability, suggesting that sucrose rather acts as a stimulatory molecule for carotenoid synthesis after the induction of synthesis has occurred (Télef et al. 2006). In considering the effects of carbohydrates, it should be kept in mind that plastids in different tissues can differ in terms of carbohydrate metabolism. For example, degradation of sucrose into fructose and glucose occurs more rapidly in tomato fruit pericarp tissue than in the placental tissue, as indicated by the increased activities of enzymes including sucrose synthase, phosphoglycerate kinase, and UDP-glucose pyrophosphorylase in the pericarp (Obiadalla-Ali et al. 2004).

## Factors influencing induction of chromoplast differentiation in fruit

### Light

Light is an important signal for the biogenesis of chloroplasts, and in the absence of light, most plants will lose the characteristic chloroplastic thylakoid membranes (Camara et al. 1995). Similarly, light is important for chromoplast differentiation, fruit colour, and carotenoid content. Although light is not strictly necessary for chromoplast development, citrus peel colouration is highly influenced by the quantity of the light received by the fruit, and this influence seems to be the same among different citrus varieties. For example, navel oranges exposed to low light illumination showed less carotenoid accumulation than fruit exposed to normal illumination (Lewis and Coggins 1964; Alquézar et al. 2008). Interestingly, carotenoid-related gene expression is not significantly correlated with changes in carotenoid content under different light intensities, suggesting that posttranscriptional regulation (for example, protein import control) might be involved in this regard.

### Temperature

In general, optimal temperature for carotenoid biosynthesis in plants is relatively low (Alquézar et al. 2008), and higher temperatures produce fruit with lower carotenoid content (Camara et al. 1995). Consistently, high temperature (exceeding 30 °C) was seen to inhibit the accumulation of lycopene in ripening tomato fruit (Brandt et al. 2006), whereas at lower temperatures (12–14 °C) citrus fruit ripening was faster and total carotenoid levels were higher (Alquézar et al. 2008). Thus, optimal temperature conditions are important for chromoplast differentiation to occur, particularly in temperature-sensitive fruits such as tomato and papaya, to ensure consistency in colour characteristics and other quality attributes.

However, papaya fruits stored at excessively low (but non-freezing) temperatures fail to ripen and suffer abnormal softening, surface pitting, and poor flavour (Wang 1989). The failure to ripen here might be connected to inefficient chloroplast-to-chromoplast transitions due to temperature stress. Use of a low storage temperature with red ripe tomatoes, which is a common practice among consumers, was shown to cause discolouration, due to lycopene degradation, and, consequently, a reduction of the nutritional properties of the fruit (Farneti et al. 2012).

### Nutrition

Nutrients play pivotal roles during chromoplast formation, particularly with regard to fruit colouration and metabolite composition. As already mentioned in earlier sections, carbon (sugars) and nitrogen were both found to be important nutritional factors controlling chromoplast development. In general, a high carbon-to-nitrogen ratio favours chromoplast differentiation, by breaking down the chloroplast structures, whereas a low ratio triggers the reverse process (Camara et al. 1995). A link between nitrogen availability and chloroplast biogenesis has also been observed, for example during leaf development in rice, where plants preferentially use limiting nitrogen resources for chloroplast maturation only during later phases of growth (Kusumi et al. 2010).

An important factor influencing the nutritional status of ripe tomato is ammonium assimilation capacity (Scarpeci et al. 2007). The activity of glutamine synthetase, the main ammonium-fixing enzyme in plants, was detected in red fruits only when plants were shifted to a highly supplemented nutritional regime. Glutamine synthetase activity can be found in chloroplasts of leaves and immature, green tomato fruit, but it disappears from chromoplasts of ripened tomatoes under normal nutrient conditions. The detection of its activity in ripe tomatoes under the supplemented regime was probably due to assimilation of excessive nitrogen and a need to fix ammonium overloaded from the vascular system (Scarpeci et al. 2007; Ferro et al. 2010). This suggests that chromoplast activity responds dynamically to the nutritional needs of the plant, and nitrogen availability changes the roles plastids must perform.

## Differentiation of chromoplasts

### Nuclear genome and plastome expression changes

During chromoplast differentiation, proteins associated with photosynthesis and starch metabolism decline in abundance, and those involved in carotenoid biosynthesis and stress responses are upregulated (Barsan et al. 2012; Wang et al. 2013; Suzuki et al. 2015). Proteins related to the biosynthesis of fatty acids, amino acids, carotenoids, vitamins, hormones, and aroma volatiles are all present in the chromoplast proteome in tomato (Barsan et al. 2010, 2012; Wang et al. 2013; Suzuki et al. 2015). These proteins are responsible for nutritional quality attributes such as colour, aroma, vitamins, and antioxidants. The presence of these proteins is of course linked to the expression of corresponding genes in the nuclear genome and the plastome.

The tomato plastome has been sequenced and found to encode 114 genes: 61 genetic system genes, comprising the transcription and translation machinery, 41 photosynthesis-related genes, and 6 other genes including that encoding the accD subunit of acetyl-CoA carboxylase involved in fatty acid biosynthesis (Kahlau et al. 2006). Because, aside from the genetic system genes, the plastome mainly encodes proteins related to photosynthesis, one would expect plastome expression to be high in chloroplasts but greatly reduced in other plastids. Historically, this was considered to be the case during the chromoplast transition, with early studies in tomato fruit ripening detecting reduced plastid ribosomal RNA and a lack of transcripts for plastome-encoded photosystem components or the large subunit of Rubisco (Piechulla et al. 1985). This was not associated with rearrangement or loss of plastid DNA (Hunt et al. 1986; Marano and Carrillo 1991), as is the case during the chloroplast-to-gerontoplast transition (Fulgosi et al. 2012).

A more comprehensive and recent transcriptomic study revealed that while plastome gene expression is indeed suppressed in mature chromoplasts, during differentiation gene expression continues, and photosynthesis-related protein expression specifically is down-regulated at the translational level (Kahlau and Bock 2008). In fact, the expression of both *rpoC2* (encoding an RNAse polymerase subunit) and *accD* (encoding a subunit of acetyl-CoA carboxylase) was seen to be up-regulated, suggesting that plastome gene expression must be maintained during differentiation for fatty acid biosynthesis (Kahlau and Bock 2008; Egea et al. 2010), to allow for the reorganization of the internal plastid membranes in advance of carotenoid synthesis (Barsan et al. 2012).

As nuclear genes encode the large majority of plastid proteins, transcriptional activity in the nucleus, translation in the cytosol, and the translocation of proteins into the plastid are all important for the assembly of the chromoplast proteome (Egea et al. 2010). Chromoplasts accumulate high levels of carotenoids, but lose photosynthetic ability, and this is reflected at the transcriptomic level. During chromoplast differentiation in tomato, nuclear genes encoding carotenoid biosynthetic proteins like PSY1 and lycopene β-cyclase (CYC-B) are up-regulated, whereas those encoding proteins involved in photosynthesis, such as the light harvesting chlorophyll *a*/*b*-binding protein (LHCP) and small subunit of Rubisco, are down-regulated (Bartley et al. 1992; Dalal et al. 2010; Pech et al. 2014). Several other genes encoding enzymes involved in the carotenoid synthesis, including DXS, phytoene desaturase, and plastid terminal oxidase associated with phytoene desaturation, are also induced dramatically during chromoplast differentiation (Fraser et al. 1994; Josse et al. 2000; Lois et al. 2000).

### Chromoplast differentiation factors

In addition to those genes responsible for the breakdown of chlorophyll (*SGR* and homologues, discussed above), several other factors have been identified as controllers of chromoplast differentiation. For example, plastid fusion and/or translocation factor (PFTF) is involved in vesicle fusion, and it remodels plastid internal membranes during the chloroplast-to-chromoplast transition in pepper (Hugueney et al., 1995). *Orange* (*Or*), a gain-of-function mutation in cauliflower, induces the leucoplasts in the floral (curd) tissue to differentiate into chromoplasts, producing orange cauliflower heads (Li et al. 2006). The mutation arrests chromoplast division specifically (chloroplasts divide normally), and induces the expression of the gene encoding PFTF.

The induction by ethylene of apetella2a (AP2a) can be positioned further upstream of the initiation of chromoplast differentiation. Disrupting the expression of *AP2a* in tomato perturbs carotenoid biosynthesis, and so the fruit becomes yellow rather than red, while the other aspects of ripening like tissue softening proceed, suggesting that the role of AP2a is in controlling chromoplast development in particular (Karlova et al. 2011). Other genes implicated in chromoplast differentiation, including the tomato homologues of *SGR*, *Or* and *PFTF*, are also down-regulated when *AP2a* expression is disrupted (Karlova et al., 2011). Thus, AP2a may represent the earliest branch of ethylene-induced ripening events specific for chromoplast differentiation.

### Hormonal regulation

The hormonal regulation of fruit ripening can be classified as either ethylene-dependent (climacteric) or ethylene-independent (non-climacteric). Common examples of climacteric fruits are tomato and most stone fruits, whereas non-climacteric fruits include citrus and pepper (Giovannoni 2004).

In climacteric fruit, the hormone ethylene induces chromoplast differentiation and other ripening events, culminating in the accumulation of metabolites responsible for food qualities such as colour, aroma, and vitamin and antioxidant content (Giovannoni 2004; Klee and Giovannoni 2011). In non-climacteric fruit, the situation appears to be different; for example, when exogenous ethylene application is used in the de-greening of citrus fruit, postharvest, its effects are limited to peel colour break, and it has no apparent effect on the internal ripening process or fruit quality (Mayuoni et al. 2011).

Much of the transcriptional reprogramming that occurs during tomato fruit ripening can be linked to the biosynthesis and perception of ethylene, as reviewed by Bapat et al. (2010). Through a signalling cascade, ethylene induces expression of the tomato homologues of *Arabidopsis* ethylene insensitive 3 (EIN3); these are termed the EIN3-like (EIL) factors (Tieman et al. 2001), and they are transcription factors that induce or repress ethylene responsive factors (ERFs). The ERFs are associated with many varied plant processes, from germination to immunity, and 19 of them are up-regulated in ripening tomato fruit, four of which (E1, E2, E4, and F2) are thought to be critical in driving the ripening process owing to their down-regulation in ripening-impaired mutants (Liu et al. 2016). Interestingly, a transcriptomic study of ripening non-climacteric pepper fruit found elements in common with the ethylene-signalling cascade in tomato; while ethylene biosynthesis is not induced in ripening pepper, downstream elements of the cascade, including the EIL homologues, are (Lee et al. 2010).

Indeed, amongst melons, examples of both climacteric and non-climacteric types may be found in closely related species, which further suggests that the two classes are not fundamentally different (Obando-Ulloa et al. 2008). Although the differences between climacteric and non-climacteric ripening are still poorly understood, the melons example suggests that non-climacteric phenotypes could arise as a result of simple mutations that uncouple ethylene synthesis and perception, rather than more fundamental differences (Obando-Ulloa et al. 2008).

Gibberellic acid can also be used to delay citrus peel colour break, as it antagonizes the expression of carotenoid biosynthetic genes and represses the accumulation of phytoene, phytofluene, and β-citraurin, which are the main carotenoids in fully ripened citrus fruit peel (Rodrigo and Zacarias 2007).

## Plastid protein import, its regulation, and involvement in plastid differentiation

### General aspects of the plastid protein import process

As already noted, during the chromoplast differentiation process, the plastid genome is essentially stable and its transcriptional activity is restricted. Thus, the build-up of chromoplasts during fruit ripening is heavily dependent upon the import of nucleus-encoded, non-photosynthetic proteins, which remodel the organellar proteome and are needed to meet the metabolic shifts and energy demands of the cells (Jarvis and López-Juez 2013; Paila et al. 2015).

Nucleus-encoded plastid proteins are synthesized in the cytosol as precursor proteins with N-terminal targeting signals called transit peptides. Transit peptides mediate the interaction of these precursor proteins with the TOC and TIC complexes that exist in the outer and inner plastid envelope membranes, respectively (Jarvis 2008). The targeting (or import) process mediated by TOC and TIC is specific, ensuring that only those proteins that are destined for the organelle gain entry, while, at the same time, avoiding mis-targeting to other cell compartments.

Identification of the components of the TOC and TIC complexes was initially achieved through the biochemical analysis of isolated pea chloroplasts (Hirsch et al. 1994; Kessler et al. 1994; Schnell et al. 1994; Wu et al. 1994; Seedorf et al. 1995; Tranel et al. 1995; Bédard and Jarvis 2005). Naturally, the TOC machinery plays critical roles in precursor protein recognition and outer membrane translocation (Jarvis 2008). The major components of the TOC apparatus are Toc159, Toc34, and Toc75 (numbers indicate molecular weight in kDa). Toc159 and Toc34 are receptor proteins with GTPase domains that regulate the recognition of precursor proteins, while Toc75 is a β-barrel protein (of the Omp85 superfamily) that forms the channel for preprotein translocation (Schnell et al. 1994; Kessler and Schnell 2002; Smith et al. 2002; Sun et al. 2002; Wallas et al. 2003; Demarsy et al. 2014; Richardson et al. 2014).

Interestingly, the TOC complex exists in multiple forms in higher plants, owing to the existence of different protein import receptor isoforms. In *Arabidopsis* (and other plants), the main isoforms of the TOC receptors (which are termed atToc159 and atToc33) have specificity for highly abundant photosynthetic precursor proteins, whereas other isoforms (i.e., atToc132/120 and atToc34) recognize non-photosynthetic and housekeeping precursor proteins (Bauer et al. 2000; Gutensohn et al. 2000; Kubis et al. 2003; Ivanova et al. 2004; Smith et al. 2004). The regulated operation of these different import pathways influences the eventual composition of the organellar proteome, and may prevent highly abundant photosynthetic proteins from outcompeting the import of equally important but less abundant housekeeping proteins. It may also control the developmental transitions of plastids—i.e., how they interconvert, from one type to another (Jarvis and López-Juez 2013).

As chromoplast development is a complex event involving many morphological and biochemical changes, it would be expected to require the reorganization of the protein import machinery. This has been demonstrated to occur during the etioplast-to-chloroplast and chloroplast-to-gerontoplast transitions in *Arabidopsis*, where the outer envelope-localized E3 ligase, SP1, selectively targets TOC complexes for ubiquitination and degradation, leading to reconfiguration of the protein import machinery (see below) (Ling et al. 2012). Interestingly, the regulator of chromoplast differentiation AP2a has been shown to up-regulate heat-shock protein 70 (Hsp70), a cytosolic chaperone associated with plastid protein import, which may be to facilitate import changes during the chromoplast transition (Karlova et al. 2011).

Downstream of the TOC complex, the TIC machinery mediates the translocation of preproteins through the inner envelope membrane with the help of various molecular chaperones (Flores-Pérez and Jarvis 2013; Nakai 2015b), some of which are proposed to meet the energetic requirements of the import process as an import motor (Shi and Theg 2013). Although several putative TIC components have been identified, the roles of some of these factors remain unclear (Jarvis and López-Juez 2013). Recently, a 1-megadalton (MDa) TIC complex containing the Tic20 protein at its core, together with several novel components (including Tic214 encoded by the plastome gene *ycf1*), was identified (Kikuchi et al. 2013). In the context of this review, it is particularly interesting to note that Tic20, like the TOC receptors, exists in multiple isoforms, and that these too have been proposed to be differentially involved in the import of photosynthetic and non-photosynthetic precursor proteins (Hirabayashi et al. 2011; Kasmati et al. 2011; Kikuchi et al. 2013; Nakai 2018). Therefore, it is conceivable that the regulated assembly of different TIC components also plays a role in the differentiation of different plastid types, including chromoplasts.

Surprisingly, the aforementioned 1 MDa TIC complex did not include those components (Tic110 and Tic40) that had been identified and studied in numerous earlier studies, leading to a degree of controversy, and fuelling questions about whether the newly identified components are directly involved in the protein import process (de Vries et al. 2015; Köhler et al. 2015; Nakai 2015a; Köhler et al. 2016; Chen and Li 2017). A possible explanation is that the 1 MDa TIC complex acts upstream with a channel-forming role, while a putative Tic110-containing motor complex acts downstream. However, a very recent study led to the discovery of a 2-MDa motor complex, comprising the protein encoded by the plastome gene *ycf2* and several nucleus-encoded FtsH-like proteins (Kikuchi et al. 2018). Regardless of the identity of the TIC machinery, upon arrival in the stroma, the transit peptide is cleaved by the stromal processing peptidase (SPP), while molecular chaperones assist with the folding of the resulting mature protein (Flores-Pérez and Jarvis 2013; Jarvis and López-Juez 2013).

### Regulation of protein import by the ubiquitin–proteasome system

The levels of TOC receptor isoforms with different preprotein recognition specificities vary developmentally depending on the biochemical requirements of the plastids (Jarvis et al. 1998; Bauer et al. 2000; Kubis et al. 2003). As noted earlier, such TOC complex rebalancing and reorganization may control the proteomic composition, developmental fate, and functions of the plastids. It has been shown that such processes are controlled, at least, in part, by the ubiquitin–proteasome system (UPS) (Ling et al. 2012; Ling and Jarvis 2015; Ling et al. 2019).

Proteolysis via the UPS involves the attachment of ubiquitin to a target protein. The attachment process is conducted by the sequential actions of three enzymes: E1 (activase), E2 (conjugase), and E3 (ligase). Once modified, a ubiquitinated protein is typically degraded by the 26S proteasome (Vierstra 2009). The targets of the UPS are identified by E3 ligases, and so, the discovery of *SP1*, which encodes a really interesting new gene (RING)-type ubiquitin E3 ligase located in the plastid outer envelope membrane generated considerable interest (Kessler 2012). Notably, it was revealed, for the first time, that the UPS regulates plastid biogenesis directly (Ling et al. 2012; Broad et al. 2016).

**Fig. 1:**
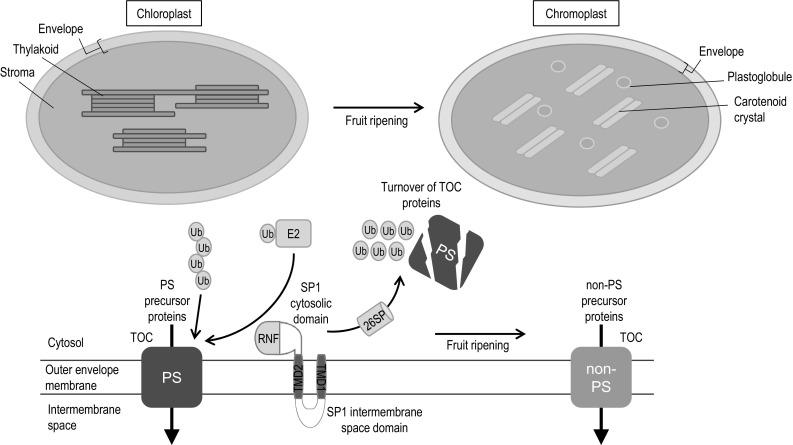
Hypothetical model showing a possible mechanism for the regulation of the chloroplast-to-chromoplast transition during fruit ripening. The chloroplasts in young green fruit are equipped with TOC complexes with specificity for precursor proteins of the photosynthetic apparatus (PS). For fruit ripening to proceed normally, metabolic shifts coupled with the down-regulation of the thylakoid biogenesis machinery and the up-regulation of carotenoid biosynthesis must occur, as part of the transitioning of chloroplasts into chromoplasts. Developing chromoplasts must import a range of different ripening-related precursor proteins, and to do this, they may require different TOC complexes with specificity for non-photosynthetic precursor proteins (non-PS). Thus, remodelling of the protein import machinery occurs during the chloroplast-to-chromoplast transition, and this may be mediated by the outer membrane E3 ligase, SP1, which targets unwanted TOC components for degradation by the 26S proteasome (26SP) in the cytosol. RNF, RING-finger domain; TMD, transmembrane domain; Ub, ubiquitin; E2, E2 conjugase

By analysing *sp1* mutant and SP1 overexpressor plants of *Arabidopsis*, Ling et al. (2012) showed that there is an inverse correlation between the SP1 expression levels and the abundance of TOC proteins; this provided strong support for the notion that SP1 is a negative regulator of the TOC machinery. Further analysis indicated that SP1 acts in the remodelling of the TOC machinery to promote the interconversion of different plastid types. In particular, SP1 was shown to have important roles in leaf senescence and de-etiolation (Ling et al. 2012). Activity of SP1 was shown to promote leaf senescence, and one may assume, therefore, that it promotes the chloroplast-to-gerontoplast conversion; this effect may be linked to TOC complex rearrangement to better accommodate the import of those proteins (e.g., catabolic enzymes) that are needed during this transition. De-etiolation, on the other hand, is characterized by the bulk import of highly abundant, photosynthesis-associated proteins, leading to major proteome changes in the plastid. For this to occur efficiently, remodelling of the TOC complex is again required: most notably, the ratio of atToc159 to atToc132/120 increases markedly in the wild type, but hardly at all in *sp1* mutant plants (Ling et al. 2012). This change is presumably to better enable the biogenesis of photosynthetic proteins. Accordingly, *sp1* mutant plants are markedly less efficient in the de-etiolation process, whereas SP1 overexpressor plants de-etiolate more efficiently than the wild type (Ling et al. 2012). Interestingly, as multiple isoforms of the TOC proteins also exist in tomato (Barsan et al. 2010), such regulation of plastid protein import by SP1 is likely to occur in this species too, and potentially in the chloroplast-to-chromoplast transition (Fig. 1).

## Future perspectives concerning the regulation of plastid transitions and implications for crop improvement

As plastids and their interconversions are important throughout plant development, the manipulation of SP1 (or of other, newly identified or yet unknown regulators of plastid biogenesis and dynamics) may enable greater control over many aspects of plant development in crops. The discovery of SP1, therefore, suggested a variety of potential applications in agriculture, possibly enabling modification of any developmental process in which plastids change type or otherwise undergo proteome reorganization (Ling et al. 2012; Ling and Jarvis 2013, 2015). Analysis of the function of SP1 in tomato, making use of transgenic plants with elevated or reduced levels of SP1 expression, may also lead to the identification of novel functions linked to the chloroplast-to-chromoplast conversion during the fruit ripening, or to other transitions.

### **Author contribution statement**

NMS wrote the initial draft of the manuscript. RGS and QL revised the initial draft and wrote additional sections. RPJ supervised the work and conducted the final editing. All authors contributed to the final manuscript.
